# Balloon occlusion of the aorta during cardiac arrest -a death blow to the intestines?

**DOI:** 10.1186/s13049-025-01321-6

**Published:** 2025-02-06

**Authors:** Bjørn Hoftun Farbu, Jostein Brede

**Affiliations:** 1 Department of Anaesthesiology and Intensive Care Medicine, St. Olav’s University Hospital, Trondheim, Norway; 2 Institute of Circulation and Medical Imaging, Faculty of Medicine and Health Sciences, Norwegian University of Science and Technology (NTNU), Trondheim, Norway; 3 Department of Research and Development, Norwegian Air Ambulance Foundation, Oslo, Norway; 4 Department of Emergency Medicine and Pre-Hospital Services, St. Olav University Hospital, Trondheim, Norway

**Keywords:** REBOA, Resuscitative endovascular balloon occlusion of the aorta, Cardiac arrest, Resuscitation, Intestinal ischaemia.

## Abstract

**Background:**

The use of Resuscitative Endovascular Balloon Occlusion of the Aorta (REBOA) in non-traumatic cardiac arrest may result in worsened intestinal ischaemia. What are the consequences?

**Main text:**

Human data on REBOA in non-traumatic cardiac arrest is limited. In general, cardiac output is reduced during resuscitation, and mesenteric blood flow may be further reduced by intravenous adrenaline (epinephrine). Balloon occlusion of the thoracic aorta will potentially lead to a complete cessation of intestinal blood flow. Experimental studies demonstrate that intestinal damage increases with REBOA inflation time, and that 45–60 min of ischaemia may result in irreversible damage. However, it is unclear when intestinal ischaemia starts to affect patient-oriented outcomes. A barrier for assessing the consequences of intestinal ischemia is that it is a challenge to diagnose. A biomarker for intestinal injury, Intestinal Fatty Acid Binding Protein (IFABP), was elevated in all cardiac arrest patients and had a striking association with mortality in one study. In another study, all patients with intestinal ischemia diagnosed on CT died. However, intestinal ischemia could be a marker of whole-body ischemia and not an independent contributor to poor outcome. The clinical importance of worsened intestinal ischemia by REBOA during cardiac arrest is not established.

**Conclusion:**

The impact of intestinal ischaemia following cardiac arrest is uncertain, but ischaemia is likely to be exacerbated by REBOA. However, inflation of the balloon will occur when the patient is still in cardiac arrest and is a means to achieve ROSC. Hence, we argue that the added intestinal ischaemia caused by REBOA may be of limited clinical importance, but this is still to be answered.

## Background

The use of Resuscitative Endovascular Balloon Occlusion of the Aorta (REBOA) in non-traumatic cardiac arrest raises concerns of intestinal ischaemia [1]. Indeed, balloon occlusion of the thoracic aorta to prioritize blood flow to the heart and brain will come at the cost of lower blood flow to the visceral organs. What are the consequences?

## Main text

Human data on REBOA in non-traumatic cardiac arrest is limited to case reports and case series [1]. Therefore, many of the effects from REBOA, including its impact on intestinal blood flow, is not established. Also in trauma care, where REBOA is used regularly, systematic evaluations of intestinal injury are lacking. During resuscitation, cardiac output is subnormal despite high quality performance [2]. Moreover, the mesenteric blood flow may be further reduced by intravenous adrenaline (epinephrine) [3, 4]. If the resuscitation is intensified by balloon occlusion of the thoracic aorta, intestinal perfusion will be even further compromised, potentially leading to complete cessation of blood flow.

A barrier for assessing the consequences of intestinal ischemia is that it is a challenge to diagnose. Clinical findings, computed tomography (CT) scans and clinically available biomarkers, all lack sensitivity and/or specificity [5]. This diagnostic challenge could lead to intestinal ischaemia not being diagnosed until autopsy [6]. An increasingly used biomarker for intestinal injury, which might prove valuable in both research and the clinic, is plasma Intestinal Fatty Acid Binding Protein (IFABP) [5].

Experimental studies give some insights into the consequences of aortic occlusion. Preclinical REBOA studies demonstrate that intestinal damage increases with inflation time [7]. Studies on humans undergoing intestinal resection on other indications than ischaemia, suggest that the intestines may regenerate after 30 min with ischaemia, whereas 45–60 min result in irreversible damage [8]. The degree of intestinal ischaemia which is tolerated before it affects clinical outcome is not known. But obviously, zero blood flow to the intestines will eventually give sufficient intestinal injury to affect patient-oriented outcomes. REBOA may potentially put the patients closer to, or beyond, this limit of tolerable intestinal ischaemia.

In patients with cardiac arrest, intestinal ischaemia is reported with various incidences. Transmural necrosis, the end-stage of intestinal ischaemia, seems to be uncommon [9]. Tam and colleagues have reported that 7% (24/363) of patients with abdominal CT had intestinal ischemia, and all these patients died [10]. We have previously shown that all cardiac arrest patients had intestinal injury, measured by IFABP. Moreover, there was a striking association between IFABP and mortality. However, IFABP was also associated with time to return of spontaneous circulation (ROSC) and lactate at admission [11]. Accordingly, if intestinal ischemia is a marker of whole-body ischemia or independently contribute to poor outcome is not established.

Intestinal ischaemia could contribute to poor outcome through an inflammatory response [12]. An inflammatory response after cardiac arrest has been shown in several studies, and the term “sepsis-like” syndrome is sometimes used [13, 14]. As the response is complex and involves many mediators, it is not easily interpreted, and associations with outcome are often reduced or not significant when adjusted in multivariable analyses [15–20]. In mediation analyses, intestinal injury following cardiac arrest did not appear to contribute to organ dysfunction through an inflammatory response [21]. Furthermore, several trials of anti-inflammatory drugs after cardiac arrest have been published, but so far only one have indicated beneficial effects on patient-oriented outcomes [22–24]. Thus, although the inflammatory response could be important for specific patients, the term “sepsis-like” syndrome might be misleading.

Irrespective of pathobiology, intestinal ischaemia after cardiac arrest remains consistently associated with increased mortality [3, 9–11, 25]. Moreover, when and how intestinal ischaemia contribute to organ dysfunction and mortality is a research priority by the European Society of Intensive Care Medicine [26]. We find that both intestinal injury biomarkers and careful application of mediation analyses are intriguing ways to improve our understanding [27].

Notably, the most common cause of death after cardiac arrest is hypoxic-ischaemic brain injury [14]. And while adrenaline could worsen microcirculation in both the brain and intestines, REBOA may increase cerebral blood flow [3, 4, 28, 29]. Additionally, it is important to acknowledge that the use of REBOA in cardiac arrest is a means to achieve ROSC, the first step towards survival. Therefore, the future use of REBOA during cardiac arrest must balance the potential benefits from increased rates of ROSC and improved cerebral blood flow against its potential risk for intestinal injury [30]. Lastly, benefits of REBOA during non-traumatic cardiac arrest is not yet demonstrated in controlled studies. The upcoming REBOARREST trial may give some indications for REBOA efficacy [1].

## Conclusion

The impact of intestinal ischaemia following cardiac arrest is uncertain, but ischaemia is likely to be exacerbated by REBOA. However, inflation of the balloon will occur when the patient is still in cardiac arrest and is a means to achieve ROSC. Hence, we argue that the added intestinal ischaemia caused by REBOA may be of limited clinical importance, but this is still to be answered.

## Data Availability

No datasets were generated or analysed during the current study.

## Abbreviations

CT: Computed tomography
IFABP: Intestinal fatty acid binding protein
REBOA: Resuscitative endovascular balloon occlusion of the aorta
ROSC: Return of spontaneous circulation
